# Effects of blood flow restriction on motoneurons synchronization

**DOI:** 10.3389/fncir.2025.1561684

**Published:** 2025-05-01

**Authors:** Mansour Taleshi, Franziska Bubeck, Leonardo Gizzi, Ivan Vujaklija

**Affiliations:** ^1^Department of Electrical Engineering and Automation, Aalto University, Espoo, Finland; ^2^Institute for Modeling and Simulation of Biomechanical Systems, University of Stuttgart, Stuttgart, Germany; ^3^Stuttgart Center for Simulation Science, University of Stuttgart, Stuttgart, Germany; ^4^Department of Biomechatronic Systems, Fraunhofer Institute for Manufacturing Engineering and Automation, Stuttgart, Germany

**Keywords:** blood flow restriction (BFR), high-density electromyography (HD-EMG), motor unit decomposition, motoneuron coherence, force tracking, isometric trapezoidal and sinusoidal contractions

## Abstract

Blood flow restriction (BFR) is a peripheral intervention that induces transient and reversible physiological perturbations. While this intervention offers a unique model to explore neuromuscular responses in multiple contexts, its impact on neural input to motoneurons remains unclear. Here, the influence of BFR on muscle force control, behavior, and neural input to motoneurons during isometric-trapezoidal and isometric-sinusoidal little finger abduction precision tasks has been studied. Sixteen healthy participants performed the tasks under pre-BFR, during BFR, and at two post-BFR conditions. High-density surface electromyography (EMG) was recorded from the abductor digiti minimi muscle, and motor unit spike trains (MUST) were decomposed using blind source separation technique. Coherence between cumulative spike trains (CSTs) of identified motor units was calculated to assess common synaptic input in the delta and alpha frequency bands. As expected, during BFR application, participants reported higher level of discomfort and significant deterioration in force-tracking performance, as measured using root mean square error (RMSE). Following the BFR release, the level of discomfort, along with impaired neuromuscular performance were reduced to pre-BFR condition. Coherence analysis revealed a prominent peak in the alpha band. The mean z-score coherence in the alpha band showed a reduction of 27% for isometric-trapezoidal and 31% for isometric-sinusoidal conditions from pre-BFR to BFR, followed by a rebound post-BFR intervention with increases of 13% and 20%, respectively. In the delta band, coherence values were consistently higher during sinusoidal tasks compared to trapezoidal ones. These findings indicate that brief BFR application led to decrease in motoneuron synchronization and force control precision likely due to desensitization as shown by changes in coherence alpha band.

## 1 Introduction

Coordinated muscle activity and precise motor control depend on the synchronized activation of motoneurons within the central nervous system (CNS) (Farmer, 1998). This synchronization is facilitated by common synaptic inputs to motoneurons, which lead to coherence in their firing patterns and enable efficient force production and movement accuracy (Baker et al., 1999). Examining coherence between various neurophysiological signals provides valuable insights into the mechanisms underlying motor coordination and the functional coupling within the CNS (Conway et al., 1995).

In terms of spectral components, the coherence signal is associated with different aspects of neural information processing. The delta band (δ-band, below 5 Hz) reflects the effective common drive to motoneurons, and represents low-frequency oscillations that synchronize motor unit firing for steady force production (De Luca and Erim, 1994). In contrast, the alpha band (α-band, 5–13 Hz) is associated with physiological tremor and proprioceptive (somatosensory) feedback, and indicates the influence of sensory inputs and spinal circuitry on motoneuron synchronization (McAuley et al., 1999).

Coherence calculation methods typically involve assessing the level of common neural drive between various neurophysiological signals, with electromyography (EMG) being a prominent example (Halliday et al., 1999). However, relying on raw EMG signals may underestimate coherence levels because motor unit action potentials are naturally filtered neural information (Farina et al., 2014). Furthermore, rectification of the EMG signal, a common practice in EMG amplitude analysis, is a non-linear operation (Clancy et al., 2002; Dideriksen et al., 2018), which can impose additional distortions by potentially altering the signal's frequency content, further complicating the interpretation of neural drive to the muscle (McGill et al., 2005). Therefore, these methods face limitations in accurately capturing the complete spectrum of neural drive.

Conversely, motor units–motor units (MUs–MUs) level coherence is performed on cumulative spike trains (CSTs) from motor units, calculated as the sum of discharge times from a selected number of motor units (Negro and Farina, 2012). This method offers a more precise measure of neural connectivity and common drive (Negro et al., 2009), and is less susceptible to noise issues that can affect neurophysiological signals.

MU–MU–based coherence can be used as a tool to understand the effect of interventions aimed at enhancing motor performance and rehabilitation outcomes (Del Vecchio et al., 2019b; Avrillon et al., 2021; Maillet et al., 2022). One such intervention is blood flow restriction (BFR), a peripheral technique involving the application of external pressure to limbs to partially restrict arterial inflow and venous outflow (Loenneke et al., 2012). BFR creates a hypoxic environment and induces transient physiological perturbations (Patterson et al., 2019). The hypoxia and metabolic stress induced by BFR can influence muscle afferent feedback and central motor drive, potentially altering motor unit recruitment strategies (Taleshi et al., 2025) and synchronization (Scott et al., 2016). These consequences of BFR present it as a valuable model to study sensorimotor system in healthy individuals.

Previous studies have demonstrated that BFR can affect EMG activity and motor unit behavior, including changes in EMG amplitude and motor unit firing rates (Yasuda et al., 2011; Gizzi et al., 2021; Bubeck et al., 2024). However, it remains unknown whether motoneurons are controlled in a similar way and receive a similar type of common synaptic input during occlusion, and how BFR administration alters synchronization within the CNS to coordinate and control muscle. Therefore, in this study, we aimed to report the changes in the common synaptic input to motoneurons in response to BFR during isometric-trapezoidal and isometric-sinusoidal precision tasks.

To achieve this, we decomposed motor unit spike trains from high-density surface electromyograms (HD-EMGs) recorded from the abductor digiti minimi (ADM) hand muscle. By calculating the coherence between groups of motoneurons at the muscle level, we sought to understand how BFR affects this neural interplay. Building upon existing evidence, our study is designed to report the direct impact of muscle BFR on force control and common synaptic input to motoneurons.

## 2 Methods

### 2.1 Participants

Sixteen participants (11 males, five females), of whom one was left-handed, all self-reporting good health with no known neurological, cardiovascular, or musculoskeletal disease, were involved in this study. Ethical approval was granted by ethics committees of University of Stuttgart (approval number 18–003) and Aalto University (decision number: D/398/03.04/2021). Participants were thoroughly informed on the study's methodology and provided informed consent by reviewing and signing the necessary documents. The average [± standard deviation (SD)] age was 32 years old (± 5 years), with an average weight of 72 kg (± 19 kg) and an average height of 173 cm tall (± 12 cm). Blood pressure measurements indicated an average systolic pressure of 112 mmHg (± 10 mmHg) and diastolic pressure of 80 mmHg (± 6 mmHg).

### 2.2 Experimental protocol and setup

Every participant completed two distinct little finger abduction movement experiments lasting ~3 h. Individual experiment consisted of either trapezoidal or sinusoidal force tracking tasks, performed in a randomized order to prevent order effects. A rest period of at least 30 min was provided between experiments.

**Figure 1:**
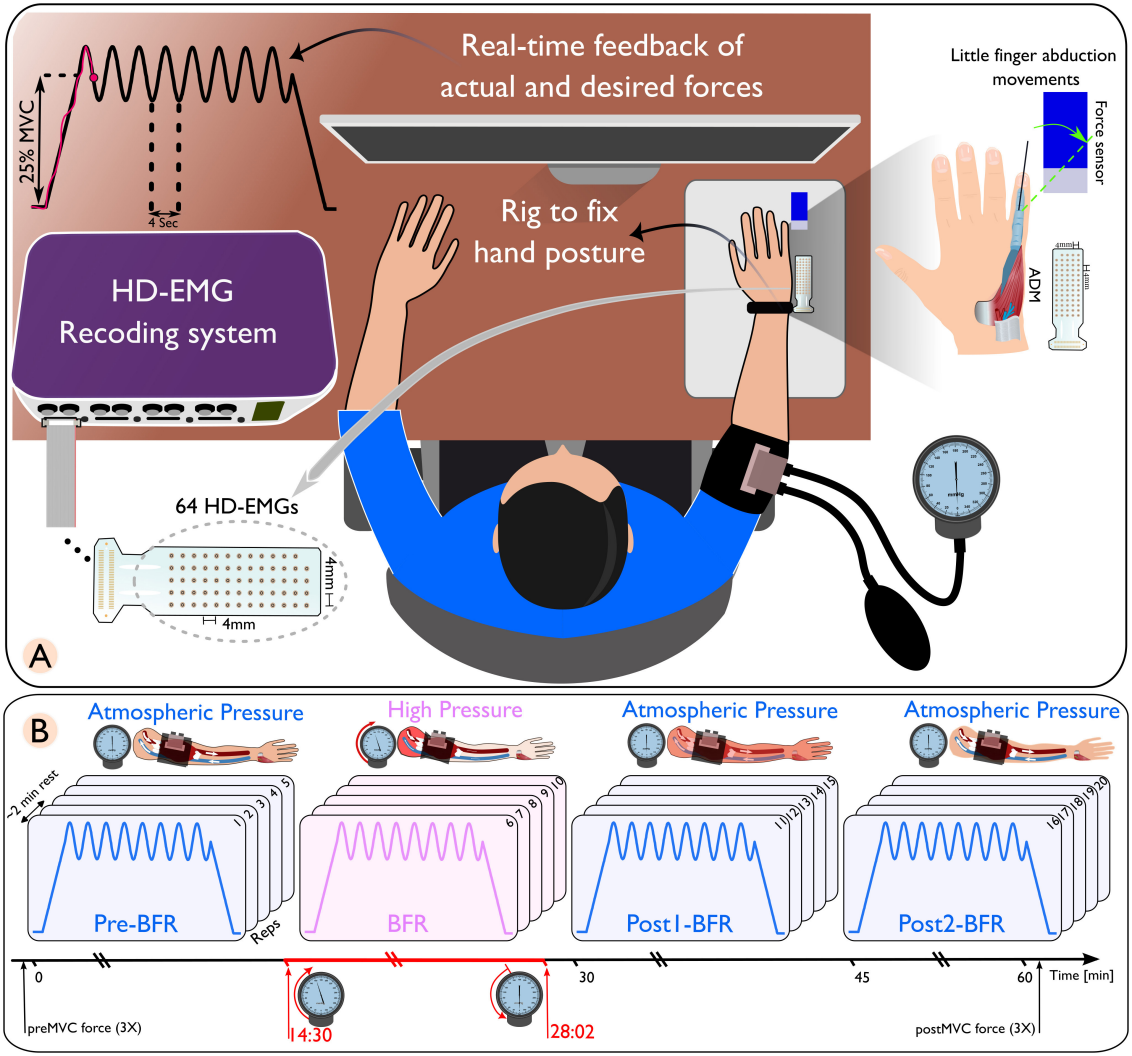
**(A)** Schematic of a high-density electromyography (HD-EMG) recording setup, featuring a subject with an arm placed in a rig to maintain hand posture, connected to a real-time feedback system displaying actual and desired force outputs. **(B)** Sequential representation of blood flow restriction (BFR) conditions on arm muscles. Only the isometric-sinusoidal experiment is shown here. The conditions are differentiated by distinct pressure changes. The pre-BFR condition, where the arm is at atmospheric pressure; the BFR condition, where high pressure is applied to restrict blood flow; followed by two post-BFR conditions where the pressure is released, returning the arm to atmospheric pressure conditions.

Participants performed three maximum voluntary contraction (MVC) force trials at the beginning (pre-MVC) and at the end (post-MVC) of each experiment (Figure 1B). During these trials, they were verbally encouraged to exert their maximum force for 3 to 5 s, with at least 120 s of rest between attempts. The highest force recorded among the three trials was used for subsequent calculations of relative force values in the motor tasks that followed. The mean (± SD) values across all participants for experiment 1, pre-MVC was 14.6 N (± 10.3 N) and post-MVC was 13.3 N (± 10.8 N), and for experiment 2, pre-MVC was 12.9 N (± 8.9 N) and post-MVC was 12.5 N (± 8.2 N).

Within each experiment, the initial five movements (pre-BFR) and the final 10 movements (post1-BFR and post2-BFR) were conducted under normal atmospheric pressure conditions, with the BFR cuff deflated. The second five repetitions (BFR) were conducted under high-pressure conditions, where the cuff pressure was elevated to 1.3 times the participant's systolic pressure (Gizzi et al., 2021). Blood pressure was measured using an analog sphygmomanometer (Boso classic and Bososcope cardio models, Bosch+Sohn, Jungingen, Germany) equipped with a cuff and stethoscope. The cuff had 32–42 cm in circumference and was placed 2.5 cm above the right elbow.

The entire BFR session lasted ~14 min, with initial recordings starting 30 s after beginning the ischemia procedure. Participants reported sensations of paresthesias immediately after cuff inflation. Within the next 5 min, they verbally indicated increasing numbness, and by ~8 min, they experienced a complete loss of touch and deep sensation in the hand and forearm. Two participants were unable to endure the entire 14 min BFR session, leading to their exclusion from further analysis.

Each isometric trapezoidal and isometric sinusoidal task was structured with a 10 s rest period, a 5 s ramp-up, a 32 s contraction phase, a 5 s ramp-down, and another 10 s rest period. The amplitude targets were set to 25% ± 5% of MVC for sinusoidal contractions and a constant 25% of MVC for trapezoidal contractions. Continuous visual feedback was provided on a display showing both the target cue and the participant's force output. This feedback enables real-time adjustments to adhere to the desired movement pattern (Figure 1A).

To standardize little finger abduction, we used a custom rig to immobilize the hand with straps. The finger was connected to a calibrated load cell (MB-50, Interface, AZ, USA). The force output was amplified 1000× (Forza-B, OT Bioelettronica) and digitized at 2,048 Hz using a Quattrocento amplifier (OT Bioelettronica).

HD-sEMG signals were recorded from the ADM muscle using GR04MM1305 electrode grids (64 channels, OT Bioelettronica, Torino, Italy) with 1 mm diameter electrodes and 4 mm inter-electrode distance. The signals were acquired at 2,048 Hz with the Quattrocento amplifier, synchronized with force data. Data collection was performed using OT Biolab+ v1.5 (OT Bioelettronica, Torino, Italy). To ensure sEMG signal quality, the participant's skin was abraded and cleaned before electrode placement. An average 7% (± 4%) of channels with significant noise were removed based on visual and spectral analysis. Data processing was done using MATLAB 2022b (MathWorks, Natick, MA, USA) and Python (Version 3.11.1) with Visual Studio Code (Microsoft Corporation, Redmond, WA, USA).

After the completion of each motor task, participants rated their perceived pain or discomfort using a modified numeric rating scale (NRS) (Gizzi et al., 2021; Bubeck et al., 2024), ranging from 1 (minimal discomfort primarily due to the experimental setup/no pain) to 10 (maximum discomfort/worst pain). NRS-10 allowed objective comparison of discomfort across individuals.

### 2.3 Data and statistical analysis

#### 2.3.1 Reported discomfort level and force tracking assessment

We analyzed the force recorded from each participant to evaluate their ability to follow the instructed force trajectory or cue. This evaluation focused on the precision or accuracy of force performance, quantified using the root mean square error (RMSE). This metric effectively captures the deviation between the desired cue and the measured force, calculated as the square root of the average of squared differences between the desired and measured force. RMSE provides a comprehensive view of the magnitude of error and offers a precise and clear picture of each participant's performance accuracy.

For each condition, we calculated the average RMSE and NRS to observe trends and deviations in performance and perceived discomfort. We plotted both RMSE and NRS variables in scatter plots to visually analyze their trend and variation across these conditions. For each condition, a 95% confidence ellipse (CE) was constructed, and its area was used to quantify the variability in the distribution of RMSE across NRS values. Given that BFR is of interest here, cluster area ratios as Pre/BFR, Post1/BFR, and Post2/BFR were calculated. These ratios provided numerical insights into how RMSE and NRS variability evolved from pre to post intervention.

#### 2.3.2 HD-EMG decomposition and motor unit tracking

After removing noisy channels, the HD-sEMG signals were decomposed into motor unit spike trains (MUST) using a convolutive blind-source separation method, as detailed by Negro et al. (2016). In summary, after removing the mean for each channel, the EMG signals were extended with an extension factor of 16 (Taleshi et al., 2022). This extension increases the ratio of the number of observations to the number of sources, enhancing the reliability, and accuracy of motor unit decomposition. Following extension, spatial whitening of the extended observation matrix was performed using a matrix obtained from its eigenvalue decomposition. Whitening is a critical step as it transforms the observed signals into a new set of signals that are uncorrelated and have unit variance, simplifying the model, and improving the efficiency of the subsequent separation process. Next, the sources were estimated by maximizing the non-Gaussianity and sparsity of the estimated sources (Negro et al., 2016). This is typically accomplished using fixed-point algorithms that iteratively refine the source estimates to maximize statistical independence. To prevent the algorithm from converging on the same source repeatedly, Gram-Schmidt orthogonalization was applied, ensuring the uniqueness of the decomposed signals. After estimating the sources, peak detection and K-means classification were utilized to separate the discharge times of the motor units from the noise (Negro et al., 2016). This process translates the signal into a spike train representation of motor unit activation.

The quality of the decomposition was then assessed using a silhouette (SIL) measure, and only motor units exhibiting a SIL value exceeding 0.85 (Taleshi et al., 2022) and a coefficient of variation (CoV) for the inter-spike interval (ISI) of <30% (*CoV*_*ISI*_ <30%) were included in subsequent analyses as detailed in (Negro et al., 2016). The SIL denotes the difference between intra-cluster and inter-cluster sums of point-to-centroid distances, normalized by dividing it by the maximum of the two computed values. This comprehensive process, from extension to quality assessment, effectively decomposes complex EMG signals into individual motor unit activities, and has been previously validated using experimental and simulated signals (Negro et al., 2016; Farina et al., 2017). After the automatic identification of the motor units, all identified MUs underwent manual inspection by carefully examining their pulse trains. Only MUs with a reliable discharge pattern were considered suitable for further analysis.

After accurately estimating MUs for each repetition of movement, we merged the MUs of five repetitions within each condition (pre-BFR, BFR, post1, and post2) into one collective set. Within each set, we removed the common units. To do so, the rate of agreement (RoA) was used to find the common units as highlighted in (Marateb et al., 2011). In summary, a pair of motor unit spike trains (*MU*_*i*_and *MU*_*j*_) was considered to match if at least 30% of their discharges were time-locked within a ± 0.5 ms window. The RoA is calculated as a ratio of the number of discharges that are common to both motor unit spike trains divided by the total number of unique discharges identified in either of the two trains, plus the number of common discharges. This is then multiplied by 100 to get a percentage. This measure effectively evaluates the degree of agreement between two spike trains and indicates how likely they are to have originated from the same motor unit based on their discharge synchrony.

#### 2.3.3 Neural input to the muscle

To estimate the level of common input at the motor unit (MU) level, we calculated the coherence between CSTs of motor units in the ADM muscle. CSTs were derived as the sum of discharge times from motor units. Coherence values range from 0 to 1, indicating the degree of correspondence between signals *x* and *y* at various frequencies. A value of 0 indicates no correlation, whereas 1 signifies perfect correlation in the frequency domain.

The magnitude-squared coherence, denoted as *C*_*xy*_(*f* ) ^=^ |*P*_*xy*_(*f* )|^2^*/*(*P*_*xx*_(*f* )*P*_*yy*_(*f* )), depends on the power spectral densities, *P*_*xx*_(*f* ) and *P*_*yy*_(*f* ), and the cross power spectral density, *P*_*xy*_(*f* ), of *x* and *y*. To assess coherence, we calculated the magnitude-squared coherence using Welchs overlapped averaged periodogram method via MATLAB's mscohere function with a 1 s Hanning window with 50% overlap.

**Figure 2:**
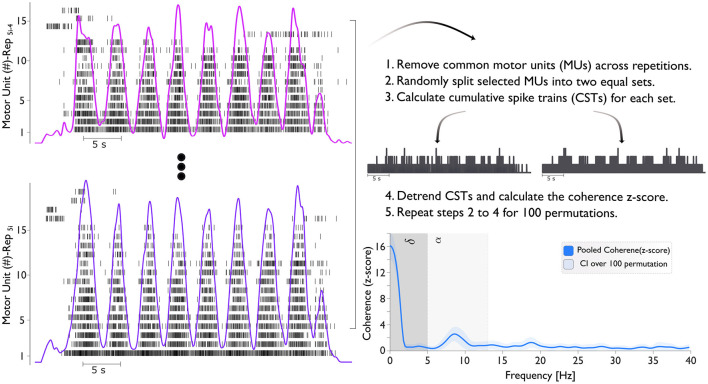
The figure illustrates the process of calculating the coherence (z-score). The left panel shows the motor unit spike train across five repetitions (Rep_5i − 4_ to Rep_5i_). Here, *i* ranges from 1 to 4, representing different conditions: pre-BFR (*i* = 1), BFR (*i* = 2), post1-BFR (*i* = 3), and post2-BFR (*i* = 4). For example, when *i* = 2, the repetitions span from *Rep*_6_ to *Rep*_10_, corresponding to the BFR condition. Cumulative Spike Trains (CSTs) are generated by summing the spike events from all MUs in each subset. Finally, the bottom right panel presents the pooled coherence (z-score) with the delta (δ) band ranges from 0–5 Hz and the alpha (α) band from 5–13 Hz.

As coherence levels increase with the number of motor neuron spike trains (Del Vecchio et al., 2019a), we considered a fixed number of MUs for both isometric-trapezoidal and isometric-sinusoidal movements to enable consistent comparisons between conditions. The count of the selected pooled motor units was determined by taking the minimum value among conditions (as shown in Figure 2).

We conducted coherence analysis on two equally sized sets of MUSTs, selecting the MUs for each set randomly. This process involved calculating the CST for each set. We repeated this procedure for all unique combinations of MUs in each group with a maximum of 100 random permutations. The resulting coherence values were averaged across these 100 combinations (Figure 2). The coherence was then converted into a standard z-score using the formula: COH_zscore_ = $\sqrt{2\text{L}}$ · atanh(COH) – bias. Here, “COH” represents coherence, “*L*” is the number of time segments used for analysis (e.g., analysis on 1 s windows with 50% overlap for 32 s of task), and “bias” is the mean “COH” z-score between 250 and 500 Hz, where no significant correlated activity is expected. This z-score transformation normalizes the variance of coherence estimates.

In this study, coherence analyses focused solely on the contraction phase (plateau/oscillation phase) of movements. We averaged coherence profiles across subjects and analyzed within two frequency bands: delta (below 5 Hz) and alpha (5–13 Hz). The mean coherence values over delta and alpha frequency bands were calculated and presented for both isometric-trapezoidal and isometric-sinusoidal movements.

#### 2.3.4 Statistical analysis

The normality of the data for each condition (pre-BFR, BFR, post1-BFR, post2-BFR) was assessed using the Kolmogorov-Smirnov test. If the normality assumption was met, paired *t*-tests were used to evaluate differences between conditions. If normality was not met, the non-parametric Wilcoxon signed-rank test was applied. Subsequently, we calculated the mean and confidence interval (CI). The CI provides a range of values within which we can estimate the true population parameter with a certain level of confidence. The formula for calculating the confidence interval is: CI = $\bar{x}$ ± *t* · *s/*$\sqrt{\text{n}}$, where CI represents the confidence interval, $\bar{x}$ is the sample mean, *t* is the critical value from the *t*-distribution corresponding to the desired confidence level, *s* is the sample standard deviation, and *n* is the sample size. In our analysis, we chose a confidence level of 95%, denoted by *t* = *t*_critical_, which allows us to assume that the true population parameter lies within the calculated confidence interval with 95% confidence. The percentage change between conditions was calculated, and a significance level of *p* < 0.05 was used to determine statistical significance. All analyses were conducted using Python's SciPy library.

## 3 Results

### 3.1 Reported discomfort level and force tracking performance assessment

**Figure 3:**
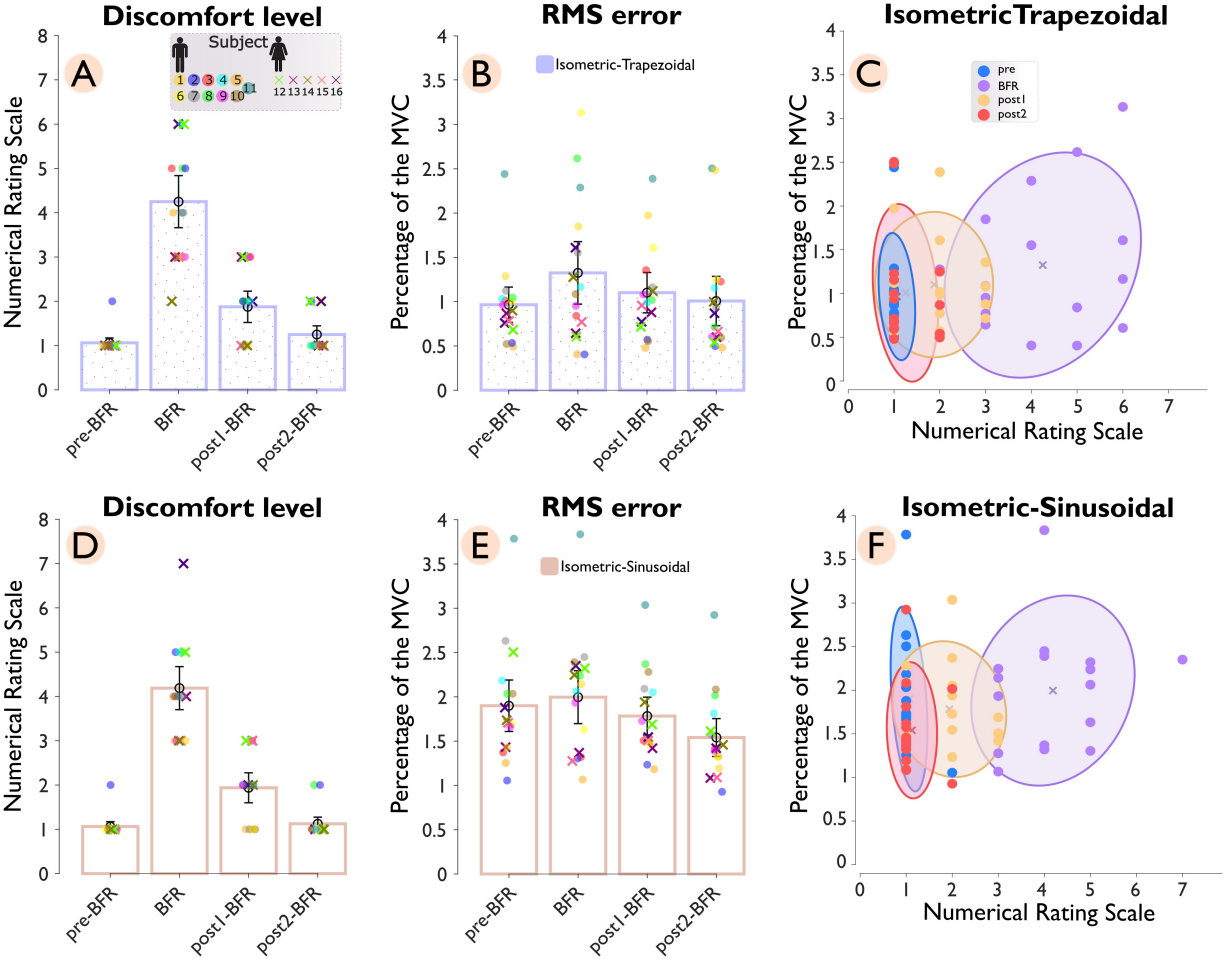
Discomfort level and tracking accuracy across conditions (pre-BFR, BFR, post1-BFR, and post2-BFR) and subjects. Panel **(A)** shows the discomfort levels for subjects during an isometric-trapezoidal task, while panel **(D)** displays the same for the isometric sinusoidal task. Each subject's data is uniquely color-coded, with female subjects denoted by “×” markers and male subjects by “*o*” markers. Panels **(B, E)** correspondingly plot the root mean square error (RMSE) for subjects across conditions for trapezoidal and sinusoidal tasks, respectively. Panels **(C, F)** provide a density distribution of discomfort levels against RMSE for trapezoidal and sinusoidal tasks, accordingly.

Figure 3 shows the reported discomfort level (Panel A and D) and tracking accuracy (Panel B and E), measured by RMSE, across pre-BFR, BFR, post1-BFR, and post2-BFR conditions for both isometric-trapezoidal and isometric-sinusoidal tasks. Each subject's data is uniquely color-coded, with female participants marked by “×” and male participants by “o”. The density distributions of discomfort levels against RMSE for trapezoidal and sinusoidal movements are shown in Panels C and F, respectively.

In the isometric-trapezoidal task, the average discomfort level showed a significant (*p* = 3.1*e*−5) increase of 300% from the pre-BFR (1.06 ± 0.13) to the BFR condition (4.25 ± 0.71). After the BFR intervention, discomfort levels dropped substantially, with a 55.88% reduction observed between the BFR and post1-BFR (1.88 ± 0.43) conditions (*p* = 2.0*e*−6) and a further 33.33% decrease between post1-BFR and post2-BFR (1.25 ± 0.24) condition (*p* = 0.015). Tracking accuracy, measured by RMSE, also declined during the BFR condition, with a 37.25% increase from pre-BFR (0.97 ± 0.24) to BFR (1.32 ± 0.43) conditions (*p* = 0.049), indicating reduced performance under BFR. The RMSE value of 1.32 ± 0.43 during the BFR condition reflects an average deviation of about 1.32% from the target force of 25% MVC. Following the BFR intervention, mean RMSE was 16.86% lower from BFR to post1BFR (1.10 ± 0.28) and 8.56% lower from post1BFR to post2BFR (1.01 ± 0.34). However, neither difference reached statistical significance (*p* = 0.168 and *p* = 0.145, respectively). Additionally, the cluster ratios with respect to the BFR condition were 0.10 for pre–BFR/BFR, 0.40 for post1-BFR/BFR, and 0.26 for post2-BFR/BFR.

In the isometric sinusoidal task, the pattern of discomfort levels showed a similar trend to the trapezoidal task, with a significant (*p* = 3.1*e*−5) increase of 294.12% from the pre–BFR (1.06 s 0.13) to the BFR' condition (4.19 ± 0.59). After the BFR intervention, discomfort levels dropped substantially, with a 53.73% reduction observed between the BFR and post1-BFR (1.94 ± 0.41) conditions (*p* = 4.0*e*−6) and a further 41.94% decrease between post1-BFR and post2-BFR (1.12 ± 0.18) condition (*p* = 0.006). Tracking accuracy, measured by RMSE, showed a minimal change during the BFR condition from pre-BFR (1.90 ± 0.35) to BFR (2.00 ± 0.36) conditions (*p* = 0.233). Following the BFR intervention, RMSE values showed a 10.68% reduction between BFR and post1-BFR (1.78 ± 0.26) condition (*p* = 0.027) and an additional 13.60% decrease between post1-BFR and post2-BFR (1.54 ± 0.26) condition (*p* = 4.6*e*−5). The cluster ratios for the sinusoidal task were 0.21 for pre-BFR/BFR, 0.50 for post1-BFR/BFR, and 0.22 for post2-BFR/BFR.

The pre-to-post comparison shows that, for the isometric-trapezoidal task, discomfort levels significantly increased from pre-BFR to post-BFR1 (*p* < 0.001) and tracking performance, as measured by RMS error, significantly deteriorated over the same interval (*p* = 0.02). In the isometric-sinusoidal task, discomfort levels were significantly increased from pre-BFR to post1-BFR (*p* < 0.001), while RMS error showed a significant improvement from pre-BFR to post2-BFR (*p* < 0.001).

### 3.2 Neural input to the muscle assessment

**Figure 4:**
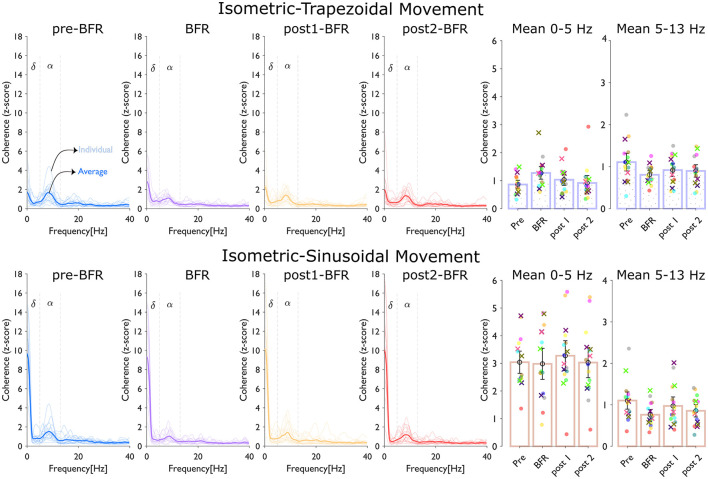
Coherence analysis across conditions (pre-BFR, BFR, post1-BFR, and post2-BFR) and subjects. The top panel, dedicated to isometric-trapezoidal movements, and the bottom panel, focusing on isometric-sinusoidal movements, both display coherence (z-score). Each line plot shows individual participant data (thin lines) and average data (thick line) across four conditions, with each condition identified by a distinct color. Adjacent to z-score line plot, bar charts show the mean z-score over the delta (δ) band (0–5 Hz) and the alpha (α) band (5–13 Hz). In each bar chart, individual subjects' data are uniquely color-coded, with females marked by “×”, and males by “*o*”.

Figure 4 shows the coherence analysis across four conditions and subjects. The top panel shows coherence (z-score) during isometric-trapezoidal movements, while the bottom panel focuses on isometric-sinusoidal movements. Within each panel, the thin lines plots show individual participant data, and the average coherence across all participants is represented by a thick line, with each condition distinguished by a unique color. Adjacent to each line plot, bar charts display the mean z-scores within the delta (δ) band (0–5 Hz) and the alpha (α) band (5–13 Hz). In these bar charts, individual subjects are color-coded, with females marked by “×” and males by “*o*”.

In the isometric-trapezoidal movements, the mean coherence in the delta band increased by 47.84% from pre-BFR (0.86 ± 0.18) to BFR (1.28 ± 0.27) condition (*p* = 0.0187). Between BFR and post1BFR (1.03± 0.25), the mean delta coherence was 18.98% lower (*p* = 0.1227), and from post1BFR to post2BFR (0.92 ± 0.32), there was an 11.35% difference (*p* = 0.2330). Neither of these differences was significant (*p* > 0.05). Coherence was 27.20% lower at BFR (0.81 ± 0.12) relative to preBFR (1.11 ± 0.26) (*p* = 0.008, significant). From BFR to post1BFR (0.92 ± 0.18), the change was 13.74% (*p* = 0.1333), and from post1BFR to post2BFR (0.90 ± 0.18), there was only a slight numerical difference (*p* = 0.7119).

In the isometric-sinusoidal movements, the delta band coherence showed a minor change from pre-BFR (3.04 ± 0.49) to BFR (2.98 ± 0.68) condition (*p* = 0.792). Between BFR and post1BFR (3.27 ± 0.66), the difference was about 9.83% (*p* = 0.277), and from post1BFR to post2BFR (3.03 ± 0.66), the change was 7.55% (*p* = 0.026), indicating statistical significance in that specific interval. In the alpha band, coherence decreased significantly by 31.00% from pre-BFR (1.10 ± 0.25) to BFR (0.76 ± 0.15) condition (*p* = 1.84*e* – 4). Between BFR (0.76 ± 0.15) and post1BFR (0.97 ± 0.26), coherence was 27.85% higher (*p* = 0.037, also significant). Lastly, from post1BFR to post2BFR (0.86 ± 0.18), the coherence difference was about 12.04% (*p* = 0.314), which was not significant.

The pre-to-post comparison shows that, for the isometric-trapezoidal task, alpha coherence significantly reduced from pre-BFR to post1-BFR (*p* = 0.013) and from pre-BFR to post2-BFR (*p* = 0.05). In the isometric-sinusoidal task, alpha coherence significantly decreased from pre-BFR to post2-BFR (*p* = 0.008).

## 4 Discussion

This study investigated the effects of BFR on motoneuron synchronization and force control during isometric-trapezoidal and isometric-sinusoidal little finger abduction tasks. By analyzing torque data, HD-sEMG signals and decomposed MUSTs, we assessed changes in force tracking performance and common synaptic input to motoneurons across pre-BFR, during BFR, and post-BFR conditions. In summary, the BFR intervention led to a noticeable increase in perceived discomfort, which was largely alleviated in the post-BFR conditions. Performance, as indicated by force-tracking accuracy, deteriorated during BFR but improved following the intervention. Similarly, coherence in the delta band tended to increase during BFR and then decreased post-intervention, while alpha band coherence decreased during BFR and partially recovered afterward.

### 4.1 Discomfort and force tracking performance

Our results confirmed a significant increase in reported discomfort levels during BFR for both isometric-trapezoidal and isometric-sinusoidal tasks, with ~300% rise compared to pre-BFR conditions. This substantial increase aligns with previous studies that reported heightened sensations of pain and discomfort under BFR conditions (Yasuda et al., 2011; Gizzi et al., 2021; Bubeck et al., 2024). The elevated discomfort can be attributed to the accumulation of metabolic by-products and reduced oxygen supply, which activate group III and IV muscle afferents and contribute to the perception of pain (Amann et al., 2010; Scott et al., 2016).

In the isometric-trapezoidal task, we observed a significant 37.25% increase in RMSE during BFR (*p* = 0.049), indicating impaired force tracking performance. This finding is consistent with previous research showing that BFR impairs isometric force steadiness and increases EMG activity due to altered sensory feedback and increased effort (Zambolin et al., 2024). The static nature of the trapezoidal task, which relies heavily on steady force production, may make it more susceptible to the negative effects of discomfort and disrupted proprioceptive feedback.

For the isometric-sinusoidal task, the RMSE showed a minimal change during BFR (*p* = 0.233). Interestingly, the initial RMSE was significantly higher in the sinusoidal movements (RMSE–pre-BFR: 1.90 ± 0.35) compared to the trapezoidal task (RMSE–pre-BFR: 0.97 ± 0.24). This higher RMSE suggests that participants found the dynamic modulation of force more challenging, possibly due to the unfamiliarity of the task and the muscle involved (the abductor digiti minimi is not typically used in daily activities requiring precise force modulation). As the task progressed, RMSE values decreased significantly post-BFR (*p* = 0.027 and *p* = 4.6*e*−5 for post1-BFR and post2-BFR, respectively), indicating improved performance likely due to motor learning and adaptation through repeated practice. This observation aligns with the notion that repetition enhances motor proficiency, especially in tasks that are initially unfamiliar or challenging (Wulf and Schmidt, 1997).

Additionally, the pairwise comparisons (pre-BFR vs. post-BFR1 and pre-BFR vs. post-BFR2) revealed that trapezoidal-task impairments in discomfort and RMS error were transient, while in the sinusoidal task, performance improved more gradually. This comparison highlights the interplay of BFR-induced discomfort and task-specific adaptations.

The cluster ratio analysis, reflecting the variability in RMSE and discomfort levels, showed the largest variability during BFR for both tasks. This supports the idea that BFR significantly impacts both subjective discomfort and objective performance measures. The reduced variability post-BFR suggests a recovery of motor control and adaptation to the task demands after the intervention.

### 4.2 Changes in common synaptic input to motoneurons

Coherence analysis of the cumulative spike trains provided insights into the changes in common synaptic input to motoneurons under BFR. We observed a significant decrease in coherence within the alpha frequency band (5–13 Hz) during BFR for both tasks with a 27.20% reduction in the trapezoidal task (*p* = 0.008) and a 31.00% reduction in the sinusoidal task (*p* = 1.84*e*−4). The alpha band is associated with proprioceptive feedback and motoneuron synchronization influenced by sensory inputs (McAuley et al., 1997, 1999). In this study, proprioceptive feedback is considered broadly to the ensemble of sensory inputs from muscle spindles (Ia and II afferents), Golgi tendon organs, joint receptors, and cutaneous afferents. While Ia afferents from muscle spindles are commonly considered the primary source in controlling low-frequency oscillations and tremor, we acknowledge that additional contributors, including secondary endings (type II afferents), tendon organs and joint receptors, can modulate motoneuron synchronization. The reduction in alpha band coherence suggests that BFR disrupts proprioceptive pathways, likely due to altered afferent feedback from reduced oxygenation and increased metabolic by-products affecting muscle spindle sensitivity (Scott et al., 2016; Zambolin et al., 2024).

Post-BFR, alpha band coherence increased in both tasks [13.74% in the trapezoidal task (*p* = 0.1333) and 27.85% in the sinusoidal (*p* = 0.037) task] indicating a partial restoration of proprioceptive feedback and motoneuron synchronization. This recovery aligns with the decrease in discomfort levels and improvements in force tracking performance, and suggests that the neuromuscular system can adapt and recover from the transient effects of BFR.

In the delta frequency band (below 5 Hz), which reflects the common drive for steady force production (De Luca and Erim, 1994), we observed different trends between the tasks. In the trapezoidal task, delta band coherence increased significantly by 47.84% during BFR (*p* = 0.0187). This suggests a compensatory mechanism where increased low-frequency common input helps maintain force output despite impaired sensory feedback and increased discomfort. Similar observations have been made in other studies, where increased central drive compensates for peripheral inhibition to sustain motor performance (Heckman and Enoka, 2012). Conversely, in the sinusoidal task, delta band coherence showed a minor change during BFR than pre-BFR. The dynamic nature of the sinusoidal task requires continuous adjustments in force output, which may rely less on low-frequency common input and more on higher-frequency inputs.

Similarly, extended pairwise testing for alpha- and delta-band coherence showed that trapezoidal alpha coherence remained suppressed through post-BFR2, whereas sinusoidal coherence shifted significantly only by the final post-BFR phase further underscoring how BFR effects and task specificity jointly influence neural synchronization.

### 4.3 Limitations and future directions

Despite the findings of this study, several limitations should be acknowledged. Our investigation focused only on the ADM muscle during specific precision tasks involving little finger abduction. This controlled condition may not fully represent the effects of BFR on larger muscle groups or during more complex, functional movements. The ADM is an isolated muscle responsible for straightforward tasks, and the neuromuscular adaptations observed may differ in muscles involved in multi-joint activities or those that function within larger muscle groups and antagonistic pairs. Incorporating multiple muscles and more complex tasks in future research could provide a more comprehensive understanding of BFR's impact on motor control and neural input. Furthermore, while BFR effectively induces muscle ischemia and activates muscle afferents, this intervention cannot isolate the effects of mechanoreceptor vs. metaboreceptor activation—it reflects a combination of both. Cuff compression may activate mechanoreceptors (Ge and Khalsa, 2003), while progressive muscle ischemia can lead to increased metabolite concentration and metaboreceptor activation (Kaufman and Hayes, 2002). Consequently, it is challenging to discern which receptor type had a greater influence on the observed changes in motoneuron synchronization and force control. Future research employing methods to separate these effects or directly measure metabolite concentrations could provide clearer insights. Additionally, we did not account for individual anatomical differences, such as arm circumference, which could influence the degree of blood flow restriction and the resulting physiological responses. Moreover, while this study was not powered or sufficiently balanced (5 females vs. 11 males) to systematically test gender-specific responses, we intend to expend our research in future in order to investigate whether gender-related differences modulate the effects of BFR on motoneuron coherence, force steadiness, and perceived discomfort. Lastly, the study focused on acute effects of BFR without considering the longitudinal impacts on neural drive and muscle function. Understanding these long-term effects is essential for determining the safety and efficacy of BFR as a therapeutic or training method. Investigating personalized occlusion pressures may allow for more targeted analyses and interventions.

### 4.4 Conclusion

The conducted experiments indicate that brief BFR application leads to altered motoneuron synchronization, which in turn negatively impacts force control. This is evidenced by impaired force-tracking performance and reduced alpha band coherence during the intervention. The partial recovery observed post-BFR includes improvements in force tracking and a restoration of motoneuron coherence. Thus, within a short period, the neuromuscular system appears to adapt to and recover from the transient effects of BFR. These findings enhance our understanding of common synaptic inputs to motoneurons under altered blood flow conditions. Future research should further investigate the long-term effects and extend these findings to broader muscle groups and functional tasks.

## Data Availability

The raw data supporting the conclusions of this article will be made available by the authors, without undue reservation.

## References

[B1] Amann M. Blain G. M. Proctor L. T. Sebranek J. J. Pegelow D. F. Dempsey J. A. (2010). Group iii and iv muscle afferents contribute to ventilatory and cardiovascular response to rhythmic exercise in humans. J. Appl. Physiol. 109, 966–976. 10.1152/japplphysiol.00462.2010 20634355 PMC2963332

[B2] Avrillon S. Del Vecchio A. Farina D. Pons J. L. Vogel C. Umehara J.. (2021). Individual differences in the neural strategies to control the lateral and medial head of the quadriceps during a mechanically constrained task. J. Appl. Physiol. 130:269–281. 10.1152/japplphysiol.00653.2020 33242302

[B3] Baker S. Kilner J. Pinches E. Lemon R. (1999). The role of synchrony and oscillations in the motor output. Exp. Brain Res. 128, 109–117. 10.1007/s002210050825 10473748

[B4] Bubeck F. Tomalka A. Siebert T. Röhrle O. Gizzi L. (2024). Altered muscle fibre activation in an antagonistic muscle pair due to perturbed afferent feedback caused by blood flow restriction. J. Electromyogr. Kinesiol. 79:102922. 10.1016/j.jelekin.2024.102922 39244815

[B5] Clancy E. A. Morin E. L. Merletti R. (2002). Sampling, noise-reduction and amplitude estimation issues in surface electromyography. J. Electromyogr. Kinesiol. 12, 1–16. 10.1016/S1050-6411(01)00033-5 11804807

[B6] Conway B. Halliday D. Farmer S. Shahani U. Maas P. Weir A.. (1995). Synchronization between motor cortex and spinal motoneuronal pool during the performance of a maintained motor task in man. J. Physiol. 489, 917–924. 10.1113/jphysiol.1995.sp021104 8788955 PMC1156860

[B7] De Luca C. J. Erim Z. (1994). Common drive of motor units in regulation of muscle force. Trends Neurosci. 17, 299–305. 10.1016/0166-2236(94)90064-7 7524216

[B8] Del Vecchio A. Falla D. Felici F. Farina D. (2019a). The relative strength of common synaptic input to motor neurons is not a determinant of the maximal rate of force development in humans. J. Appl. Physiol. 127, 205–214. 10.1152/japplphysiol.00139.2019 31120812

[B9] Del Vecchio A. Germer C. Elias L. Fu Q. Fine J. Santello M.. (2019b). The human central nervous system transmits common synaptic inputs to distinct motor neuron pools during nonsynergistic digit actions. J. Physiol. 597, 5935–5948. 10.1113/JP278623 31605381 PMC6972516

[B10] Dideriksen J. L. Negro F. Falla D. Kristensen S. R. Mrachacz-Kersting N. Farina D. (2018). Coherence of the surface emg and common synaptic input to motor neurons. Front. Hum. Neurosci. 12:207. 10.3389/fnhum.2018.00207 29942254 PMC6004394

[B11] Farina D. Merletti R. Enoka R. M. (2014). The extraction of neural strategies from the surface emg: an update. J. Appl. Physiol. 117, 1215–1230. 10.1152/japplphysiol.00162.2014 25277737 PMC4254845

[B12] Farina D. Vujaklija I. Sartori M. Kapelner T. Negro F. Jiang N.. (2017). Man/machine interface based on the discharge timings of spinal motor neurons after targeted muscle reinnervation. Nature Biomed. Eng. 1:0025. 10.1038/s41551-016-0025

[B13] Farmer S. (1998). Rhythmicity, synchronization and binding in human and primate motor systems. J. Physiol. 509, 3–14. 10.1111/j.1469-7793.1998.003bo.x 9547376 PMC2230956

[B14] Ge W. Khalsa P. S. (2003). Encoding of compressive stress during indentation by group iii and iv muscle mechano-nociceptors in rat gracilis muscle. J. Neurophysiol. 89, 785–792. 10.1152/jn.00624.2002 12574456

[B15] Gizzi L. Yavuz U. S. Hillerkuss D. Geri T. Gneiting E. Domeier F.. (2021). Variations in muscle activity and exerted torque during temporary blood flow restriction in healthy individuals. Front. Bioeng. Biotechnol. 9:557761. 10.3389/fbioe.2021.557761 33816445 PMC8017222

[B16] Halliday D. M. Conway B. A. Farmer S. F. Rosenberg J. R. (1999). Load-independent contributions from motor-unit synchronization to human physiological tremor. J. Neurophysiol. 82, 664–675. 10.1152/jn.1999.82.2.664 10444664

[B17] Heckman C. Enoka R. M. (2012). Motor unit. Comprehensive physiology, (4):2629–2682. 10.1002/j.2040-4603.2012.tb00465.x 23720261

[B18] Kaufman M. P. Hayes S. G. (2002). The exercise pressor reflex. Clin. Auton. Res. 12, 429–439. 10.1007/s10286-002-0059-1 12598947

[B19] Loenneke J. P. Abe T. Wilson J. M. Ugrinowitsch C. Bemben M. G. (2012). Blood flow restriction: how does it work? Front. Physiol. 3:392. 10.3389/fphys.2012.00392 23060816 PMC3463864

[B20] Maillet J. Avrillon S. Nordez A. Rossi J. Hug F. (2022). Handedness is associated with less common input to spinal motor neurons innervating different hand muscles. J. Neurophysiol. 128, 778–789. 10.1152/jn.00237.2022 36001792

[B21] Marateb H. R. McGill K. C. Holobar A. Lateva Z. C. Mansourian M. Merletti R. (2011). Accuracy assessment of ckc high-density surface emg decomposition in biceps femoris muscle. J. Neural Eng. 8:066002. 10.1088/1741-2560/8/6/066002 21975280 PMC3422671

[B22] McAuley J. Farmer S. Rothwell J. Marsden C. (1999). Common 3 and 10 hz oscillations modulate human eye and finger movements while they simultaneously track a visual target. J. Physiol. 515, 905–917. 10.1111/j.1469-7793.1999.905ab.x 10066915 PMC2269180

[B23] McAuley J. Rothwell J. Marsden C. (1997). Frequency peaks of tremor, muscle vibration and electromyographic activity at 10 hz, 20 hz and 40 hz during human finger muscle contraction may reflect rhythmicities of central neural firing. Exp. Brain Res. 114, 525–541. 10.1007/PL00005662 9187289

[B24] McGill K. C. Lateva Z. C. Marateb H. R. (2005). Emglab: an interactive emg decomposition program. J. Neurosci. Methods 149, 121–133. 10.1016/j.jneumeth.2005.05.015 16026846

[B25] Negro F. Farina D. (2012). Factors influencing the estimates of correlation between motor unit activities in humans. PloS ONE 7:e44894. 10.1371/journal.pone.0044894 23049762 PMC3458041

[B26] Negro F. Holobar A. Farina D. (2009). Fluctuations in isometric muscle force can be described by one linear projection of low-frequency components of motor unit discharge rates. J. Physiol. 587, 5925–5938. 10.1113/jphysiol.2009.178509 19840996 PMC2808549

[B27] Negro F. Muceli S. Castronovo A. M. Holobar A. Farina D. (2016). Multi-channel intramuscular and surface emg decomposition by convolutive blind source separation. J. Neural Eng. 13:026027. 10.1088/1741-2560/13/2/026027 26924829

[B28] Patterson S. D. Hughes L. Warmington S. Burr J. Scott B. R. Owens J.. (2019). Blood flow restriction exercise: considerations of methodology, application, and safety. Front. Physiol. 10:533. 10.3389/fphys.2019.00533 31156448 PMC6530612

[B29] Scott B. R. Loenneke J. P. Slattery K. M. Dascombe B. J. (2016). Blood flow restricted exercise for athletes: A review of available evidence. J. Sci. Med. Sport 19, 360–367. 10.1016/j.jsams.2015.04.014 26118847

[B30] Taleshi M. Bubeck F. Brunner P. Gizzi L. Vujaklija I. (2025). Observing changes in motoneuron characteristics following distorted sensorimotor input via blood flow restriction. J. Appl. Physiol. 138, 559–570. 10.1152/japplphysiol.00603.2024 39813017

[B31] Taleshi M. Yeung D. Negro F. Vujaklija I. (2022). Muscle synergy-driven motor unit clustering for human-machine interfacing. Annu. Int. Conf. IEEE Eng. Med. Biol. Soc. 2022, 4147–4150. 10.1109/EMBC48229.2022.9871356 36086401

[B32] Wulf G. Schmidt R. A. (1997). Variability of practice and implicit motor learning. J. Exp. Psychol. Learn. Memory Cogn. 23:987. 10.1037//0278-7393.23.4.987

[B33] Yasuda T. Ogasawara R. Sakamaki M. Ozaki H. Sato Y. Abe T. (2011). Combined effects of low-intensity blood flow restriction training and high-intensity resistance training on muscle strength and size. Eur. J. Appl. Physiol. 111, 2525–2533. 10.1007/s00421-011-1873-8 21360203

[B34] Zambolin F. Duro Ocana P. Goulding R. Sanderson A. Venturelli M. Wood G.. (2024). The corticomuscular response to experimental pain via blood flow occlusion when applied to the ipsilateral and contralateral leg during an isometric force task. Psychophysiology 61:e14466. 10.1111/psyp.14466 37872004

